# Supplementary material for: Transplantation of Fibroblast Sheets with Blood Mononuclear Cell Culture Exerts Cardioprotective Effects by Enhancing Anti-Inflammation and Vasculogenic Potential in Rat Experimental Autoimmune Myocarditis Model
Source: Biology (Basel). 2022 Jan 10;11(1):106. doi: 10.3390/biology11010106 (PMC8772944; doi:10.3390/biology11010106)

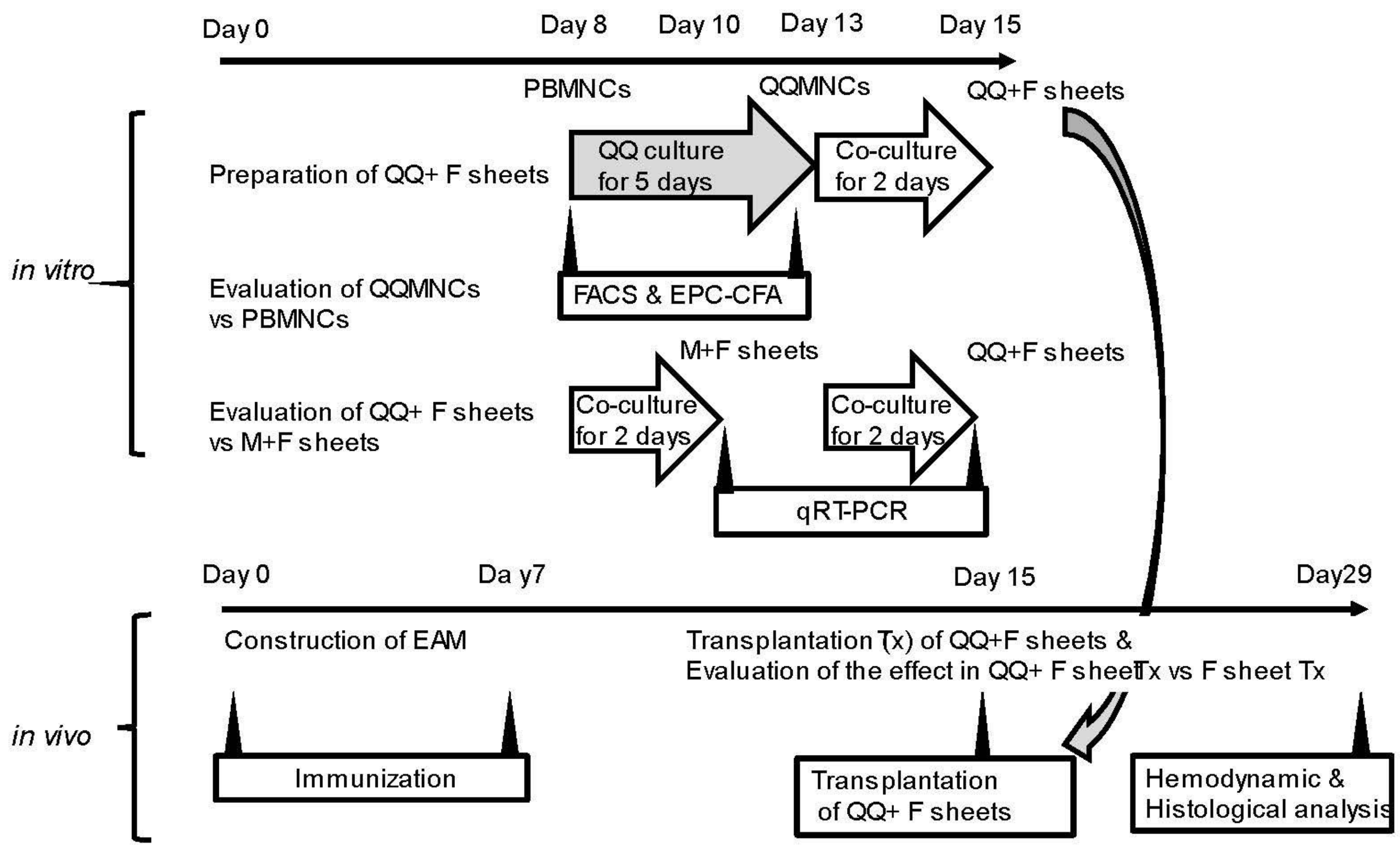

Figure S1. Experimental protocol. Evaluated items and date of the schedule are shown.

Table S1. Contents of QQ culture medium and semisolid culture for EPC colony formation assay.

QQ culture medium	Company, Catalog No.	Final Concentration
Stem line® II Hematopoietic Stem Cell Expansion Medium	Sigma-Aldrich, #S0192	2ml/dish
Recombinant Rat SCF	Peprotec, #AF400-22	100 ng/ml
Recombinant Murine Flt-3 ligand	Peprotec, #250-31L	100 ng/ml
Recombinant Rat TPO	Peprotec, #400-34	20 ng/ml
Recombinant Rat VEGF	Peprotec, #400-31	50 ng/ml
Recombinant RatIL-6	Peprotec, #400-06	20 ng/ml

Semisolid culture medium		
MethoCult SFTM	STEMCELL Technologies, #M3236	
Recombinant Rat SCF	Peprotec, #AF400-22	100ng/ml
Recombinant Rat VEGF	Peprotec, #400-31	50 ng/ml
Recombinant Rat basic FGF	Peprotec, #400-29	50 ng/ml
Recombinant Rat EGF	Peprotec, #AF400-25	50 ng/ml
Recombinant Rat IGF-1	Peprotec, #250-19	50 ng/ml
Recombinant Murine IL-3	Peprotec, #213-13	20 ng/ml
Heparin	Ajinomoto Pharma	2 IU/ml
FBS	SAFC Bioscience, #12303	10% (vol/vol)

Table S2. Flow cytometric analysis.

**Stain No.1**					
**Antibody**	Fluorescence	2nd antibody	Isotype		Company, Catalog No.
**CD34**		anti-sheep IgG Alexa594: invitrogen #A-11016	sheep	IgG1	R&D, #AF6518
**CD133**		anti-rabbit IgG BV421:BD #585014	Rabbit	IgG	MyBioSourse, #MBS176043
**KDR**	DyLight 488		Rabbit	IgG	Abnova, #PAB15088
**CD3**	PerCP-eFluro710		mouse	IgG3	Bioscience, # 46-0030
**CD11b/c**	PE-Cy7		mouse	IgG2a	BD Pharmingen™, #562222
**CD25 biotin**		Stv Alexa488: biolegend #405235	mouse	IgG1	Bioscience, #13-0390-81
**CD31**	eFluro660		mouse	IgG1	Bioscience, #310
**CD45**	APC-eFluro780		mouse	IgG1	Bioscience #47-0461

**Stain No.2**					
**Antibody**	Fluorescence	2nd antibody	Isotype		Company, Catalog No.
**CD3**	PerCP-eFluro710		mouse	IgG3	Bioscience, # 46-0030
**CD 4**	PE		mouse	IgG2a	BioLgend, #TD-203307
**CD 8**	Alexa 647		mouse	IgG1	BioLgend, #TD-20170
**CD25 biotin**		Stv Alexa488: biolegend 405235	mouse	IgG1	Bioscience, #13-0390-81

**Stain No.3**					
**Antibody**	Fluorescence	2nd antibody	Isotype		Company, Catalog No.
**CD68**	PE-Cy7		human	IgG	miltenyibiotec, #130-102-726
**CD163**		anti-mouse IgG APC: biolegend 405308	mouse	IgG1	GNT, #GTX４２３６７
**CD206**	PE		human	IgG	Bioss, #bs-2664R-PE

Table S3. List of primers.

Gene name	Abbrev.	Another name		Sense primer	Antisense primer
Il-10			TaqMan (Invitrogen), #Rn00563409_m1	5′-CAGACCCACATGCTCCGAGA-3′	5′-CAAGGCTTGGCAACCCAAGTA-3′
Foxp3			TaqMan (Invitrogen)#Rn01525092_m1	5′-CCCAGGAAAGACAGCAACCTT-3′	5′-CTGCTTGGCAGTGCTTGAGAA-3′
18S ribosomal RNA			TaqMan	5′-GGGTCATAAGCTTGCGTTGATTAAG-3′	5′-TCCGAGGGCCTCACTAAACC-3′
Bnp			SYBR Green	5′-CTGGGAAGTCCTAGCCAGTCT-3′	5′-GTCTATCTTCTGCCCAAAGCAG-3′
**18S ribosomal RNA**	*18SrRNA*		SYBR Green	5′-TGCCAGAGTCTCGTTCGTTA-3′	5′-GGTGCATGGCCGTTCTTA-3′
**Hepatocyte growth factor**	*Hgf*		SYBR Green	5′-GCCATGAATTTGACCTCTATGA-3′	5′-CTTAGTGATGGATACTGTCCCCTTA-3′
**Insulin like growth factor 1**	*Igf1*		SYBR Green	5′-TGCTTGCTCACCTTTACCAG-3′	5′-ATAGCCTGTGGGCTTGTTGA-3′
**Interleukin 1 beta**	*Il-1β*		SYBR Green	5′-TGTGATGAAAGACGGCACAC-3′	5′-CTTCTTCTTTGGGTATTGTTTGG-3′
**Interleukin- 10**	*Il-10*		SYBR Green	5′-AGTGGAGCAGGTGAAGAATGA-3′	5′-TCATGGCCTTGTAGACACCTT-3′
**Interleukin- 17**	*Il-17*		SYBR Green	5′-CCTCAACCGTTCCACTTCAC-3′	5′-CCTCAGCGTTGACACAGC-3′
**Matrix metallopeptidase 9**	*Mmp9*		SYBR Green	5′-CCTCTGCATGAAGACGACATAA-3′	5′-GGTCAGGTTTAGAGCCACGA-3′

**Matrix metallopeptidase 2**	*Mmp2*		SYBR Green	5′-GCACCACCGAGGATTATGAC-3′	5′-CACCCACAGTGGACATAGCA-3′
**Tumor necrosis factor**	*Tnf*	*Tnf-α*	SYBR Green	5′-TGAACTTCGGGGTGATCG-3′	5′-GGGCTTGTCACTCGAGTTTT-3′
**Vascular endothelial growth factor a**	*Vegf-a*		SYBR Green	5′-CGGATCAAACCTCACCAAAGC-3′	5′-GGTCTGCATTCACATCTGCTATG-3′
**Vascular endothelial growth factor b**	*Vegf-b*		SYBR Green	5′-CTGCAGCTGGCCTGTACC-3′	5′-TGGCACGTGCATAAACATCT-3′
**Mannose receptor c-type 1**	*Cd206*	*Mrc1*	SYBR Green	5′-AACAACAGGCAGGAGGACTG-3′	5′-GCTCTATCTGCCCAGTATCCA-3′

Table S4. Percentage of positive cell populations in QQMNCs and PBMNCs.

Healthy control	% in PBMNCs	% in QQMNCs	Ratio	*P*-value
CD34	3.43 ± 0.87	5.56 ± 1.87	1.38 ± 0.27*	0.0400
CD133	0.34 ± 0.03	0.14 ± 0.07	0.55 ± 0.26	0.055
CD4+/CD3+	79.67 ± 2.29	88.02 ± 2.03	1.10 ± 0.15	0.055
CD8+/CD3+	88.02 ± 1.68	51.44 ± 0.50	0.27 ± 0.01**	0.002
CD31	18.79 ± 2.00	5.14 ± 0.79	1.01 ± 0.16	0.818
VEGFR-2/CD34	0.36 ± 0.13	1.65 ± 0.61	5.93 ± 1.05	0.18
CD163	1.52 ± 0.48	11.85 ± 2.55	12.68 ± 3.49*	0.040
CD206+CD68	1.00 ± 0.01	10.20 ± 1.35	10.20 ± 1.35**	0.002
CD11b+CD11c	6.94 ± 3.57	9.43 ± 2.31	1.36 ± 0.47	0.485
CD25/CD3+CD4	4.24 ± 0.09	4.92 ± 0.02	1.16 ± 0.10	0.631

Myocarditis	% in PBMNCs	% in QQMNCs	Ratio	*P*-value
CD34	1.00 ± 0.27	3.42 ± 1.36	3.42 ± 0.14	0.011
CD133	0.10 ± 0.03	0.22 ± 0.14	2.18 ± 0.58	0.818
CD4+/CD3+	67.38 ± 8.00	9.23 ± 1.34	1.45 ± 0.16*	0.015
CD8+/CD3+	11.57 ± 0.84	6.6 ± 0.40	0.60 ± 0.07	0.002
CD31	18.79 ± 0.80	98.85 ± 0.27	5.26 ± 0.11**	0.002
VEGFR-2/CD34	1.00 ± 0.19	1.88 ± 1.48	1.88 ± 0.37*	0.036
CD163	1.00 ± 0.16	10.85 ± 6.00	28.34 ± 5.65**	0.002
CD206+CD68	1.00 ± 0.01	9.82 ± 5.59	19.29 ± 4.98**	0.002
CD11b+CD11c	13.43 ± 1.16	1.33 ± 0.51	0.12 ± 0.05**	0.002
CD25/CD3+CD4	5.00 ± 2.89	6.33 ± 4.07	1.27 ± 0.27	0.476

Table S5. qRT-PCR analysis of PBMNCs and QQMNCs derived from EAM rats.

	Vegf-a	*Vegf-b*	*Hgf*	*Igf-1*	*Cd206 (Mrc1)*	*Il-10*	*Foxp3*	*Mmp2*	*Mmp9*	*Il-17*	*Tnf*	*Il-1β*
PBMNVs	1 ± 0.20	1 ± 0.62	1 ± 0.13	1 ± 0.45	1 ± 0.41	1 ± 0.39	1 ± 0.41	1 ± 0.41	1 ± 0.42	1 ± 0.48	1 ± 0.49	1 ± 0.61
QQMNCs	4.12 ± 0.88**	56.08 ± 3.58**	3.58 ± 0.44	2.34 ± 1.06	4.75 ± 1.94*	0.11 ± 0.04**	0.21 ± 0.09	140.66 ± 57.42**	1.03 ± 0.21	34.54 ± 2.15**	4.12 ± 2.42**	0.20 ± 0.05
P value	0.009	0.002	0.093	0.349	0.015	0.009	0.24	0.002	0.528	0.002	0.009	0.132

Data are shown as mean ± SE. N = 5 or 6, *P < 0.05, **P < 0.01 vs QQMNC group.

Table S6. qRT-PCR analysis in cell sheets.

	Vegf-a	*Vegf-b*	*Hgf*	*Igf-1*	*Cd206 (Mrc1)*	*Il-10*	*Foxp3*	*Mmp2*	*Mmp9*	*Il-17*	*Tnf*	*Il-1β*
**F sheet**	1 ± 0.14	1 ± 0.15	1 ± 0.07	1 ± 0.14	1 ± 0.04	1 ± 0.27	1 ± 0.32	1 ± 0.20	1 ± 0.19	1 ± 0.38	1 ± 0.13	1 ± 0.34
**M + F sheet**	1.28 ± 0.33	0.94 ± 0.17	1.40 ± 0.31	0.92 ± 0.22	2.01 ± 0.44	1.09 ± 0.12	3.82 ± 1.01	1.33 ± 0.27	1.40 ± 0.32	1.14± 0.40	3.52± 1.03	3.11 ± 0.62
**QQ + F sheet**	1.40 ± 0.46	1.73 ± 0.11*†	2.25 ± 0.24*	1.55 ± 0.18	2.85 ± 0.19*	2.44 ± 0.43*	0.98 ± 0.29	1.59 ± 0.36	1.90 ± 0.49	0.85 ± 0.39	2.42 ± 0.13	5.15 ± 0.81**
***P* Value**	0.760	0.008	0.032	0.108	0.024	0.029	0.058	0.616	0.325	0.065	0.077	0.009

N = 5 for each group. **P* < 0.05; ***P* < 0.01 versus F sheet, †*P* < 0.05; ††P < 0.01 versus M + F sheet. QQMNCs were obtained from a rat model of FM.

Table S7. Hemodynamic indices 2 weeks after operation.

Basics hemodynamics	Sham	F sheet	QQ+F Sheet	Healthy control	*P* value
EDPVR on pressure-load	475 ± 132	405 ± 81	176 ± 28	100 ± 6.4*†	0.001
ESPVR on pressure-load	1783 ± 214	1709 ± 270	1263 ± 168	1017 ± 111*	0.042
Heart rate (bpm)	315 ± 15	313 ± 10	312 ± 15	356 ± 11	0.053
Ejection fraction (%)	51 ± 6	42 ± 4	37 ± 7	41 ± 2	0.616
Cardiac output (ml/min)	27 ± 4	26 ± 2	37 ± 5	62 ± 7*†	0.042
End-systolic pressure (mmHg)	101 ± 10	100 ± 7	104 ± 8	93 ± 9	0.879
End-diastolic pressure (mmHg)	17 ± 5	8 ± 2	11 ± 2	13 ± 2	0.346
dP/dt max (mmHg/s)	6389 ± 803	7214 ± 798	7271 ± 905	6354 ± 714	0.693
−dP/dt min (mmHg/s)	4747 ± 704	4436 ± 463	5463 ± 563	5324 ± 858	0.711
τ (ms)	14 ± 1	13 ± 1	12 ± 1	12 ± 1	0.528

N = 6 for each group. *P < 0.05; versus sham, †P < 0.05; versus F sheet.

Figure S2. The graphs of hemodynamic indices 2 weeks after operation.

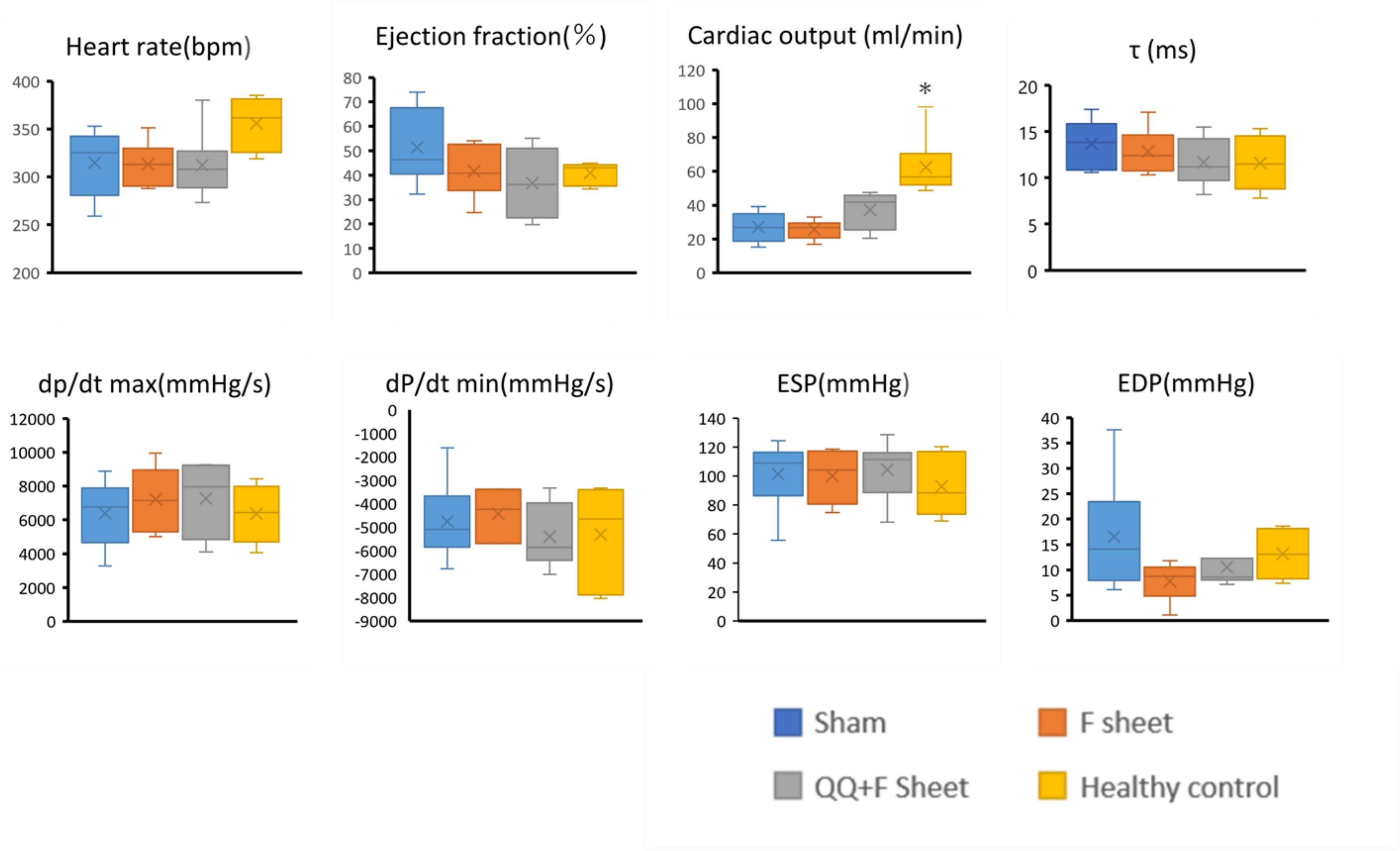

Supplement: Supplementary file 1 [file biology-11-00106-s001.zip › biology-1484485-supplementary.pdf]
